# NLRP3 inflammasome activation mediates radiation-induced pyroptosis in bone marrow-derived macrophages

**DOI:** 10.1038/cddis.2016.460

**Published:** 2017-02-02

**Authors:** Yan-gang Liu, Ji-kuai Chen, Zi-teng Zhang, Xiu-juan Ma, Yong-chun Chen, Xiu-ming Du, Hong Liu, Ying Zong, Guo-cai Lu

**Affiliations:** 1 Department of Health Toxicology, College of Tropical Medicine and Public Health, Second Military Medical University, Shanghai 200433, China; 2 Radiology Department X-ray Room, The Second People's Hospital, Neijiang, Sichuan 641100, China; 3 CTI Biotechnology (Suzhou) Co., Ltd, Jiangsu 215300, China

## Abstract

A limit to the clinical benefit of radiotherapy is not an incapacity to eliminate tumor cells but rather a limit on its capacity to do so without destroying normal tissue and inducing inflammation. Recent evidence reveals that the inflammasome is essential for mediating radiation-induced cell and tissue damage. In this study, using primary cultured bone marrow-derived macrophages (BMDM) and a mouse radiation model, we explored the role of NLRP3 inflammasome activation and the secondary pyroptosis underlying radiation-induced immune cell death. We observed an increasing proportion of pyroptosis and elevating Caspase-1 activation in 10 and 20 Gy radiation groups. *Nlrp3* knock out significantly diminished the quantity of cleaved-Caspase-1 (p10) and IL-1*β* as well as the proportion of pyroptosis. Additionally, *in vivo* research shows that 9.5 Gy of radiation promotes Caspase-1 activation in marginal zone cells and induces death in mice, both of which can be significantly inhibited by knocking out *Nlrp3*. Thus, based on these findings, we conclude that the NLRP3 inflammasome activation mediates radiation-induced pyroptosis in BMDMs. Targeting NLRP3 inflammasome and pyroptosis may serve as effective strategies to diminish injury caused by radiation.

Radiotherapy is used extensively with bone marrow transplants and for restricting the growth and spread of a variety of tumors such as prostate cancer, lung cancer, and renal-cell carcinoma, etc.^1, 2, 3^ A limit to the clinical benefit of radiotherapy is not an incapacity to eliminate neoplastic cells but rather a limit on its capacity to do so without destroying normal tissue and inducing inflammation.^4^ Although the principal pathogenesis of radiation for damaging normal tissue is through the depletion of rapidly dividing cells, such as hematopoietic cells, vascular endothelial cells and somatic stem cells, emerging concepts suggest that exposure to radiation can also result in the activation and damage of peripheral immune cells and pro-inflammatory cytokine release, which in turn may impair the recovery and repopulation of destroyed cells and even result in a systemic response syndrome.^4, 5, 6^

Macrophages are recruited as a first response to radiation-induced damage.^7^ Alterations in macrophages following radiation have been observed during both early and late phases of tissue injury.^8^ Radiation polarizes macrophages towards an M1 phenotype, which is known as the pro-inflammatory phenotype, and enhances the secretion of M1 cytokines such as TNF-α, IL-12 and IFN-*γ*, which participate in pro-inflammatory responses.^9, 10^ Activated M1 macrophages then promote extracellular matrix destruction, cell apoptosis, chronic inflammation (fibrosis) and tissue injury.^4^ Additionally, Raj *et al.* found that *N*-acetyl tryptophan glucoside (NATG) pretreatment overcomes the radiation-induced immune response by affecting macrophages and thus contributes to radioprotection.^11^ Thus, to explore the mechanism of radiation-induced immune cell, particularly macrophage, activation or damage, it is of paramount importance to find ways to limit the side effects of radiation and to maximize future therapeutic benefit. In the present study, the mechanism of immune system damage under radiation was studied using primary cultured bone marrow-derived macrophages (BMDMs).

Mainly described in macrophages and dendritic cells, pyroptosis is recognized as a model of cell death distinct from apoptosis and is thought of as a regulated form of necrosis.^12, 13^ Unlike apoptosis, pyroptosis is initiated by the recognizing of NOD-like receptors (NLRs) to pathogen-associated molecular patterns (PAMPs) and danger-associated molecular patterns (DAMPs), leading to the assembly of a large multiprotein complex termed inflammasome which recruits and cleaves the precursor of Caspase-1 (pro-Caspase-1, p45) to its active form.^14, 15^ Active Caspase-1 then causes the enzymolysis of the precursors for inflammatory cytokines IL-1*β* and IL-18 or induces pore formation on the plasma membrane, which results in cell swelling and the release of cytosolic contents such as lactate dehydrogenase (LDH) and pro-inflammatory cytokines.^16, 17^ However, the role of pyroptosis in radiation-induced macrophage damage remains unclear.

In recent years, NLRP3 inflammasome upregulation at the expression or activation level has been reported to play an important role in radiation-induced lung inflammation, oral mucositis and skin lesions.^18, 19, 20, 21^ Activation of Caspase-1 has also been linked with radiation exposure in the immune cells from spleen or hippocampal neural stem cells.^5, 22^ Furthermore, Stoecklein *et al.* demonstrated that inflammasome activation occurs in many immune cell types following radiation exposure.^5^ As Caspase-1 was found to be activated by radiation in many studies, whether the components downstream of active Caspase-1, such as pyroptosis, play a role in radiation-induced immune cell death remains unknown. In this study, using primary cultured BMDMs and a mouse radiation model, we reported here for the first time that NLRP3 inflammasome-mediated pyroptosis is of great significance in radiation-induced BMDM death.

## Results

### Radiation induced pyroptosis in bone marrow-derived macrophages

**Figure 1:**
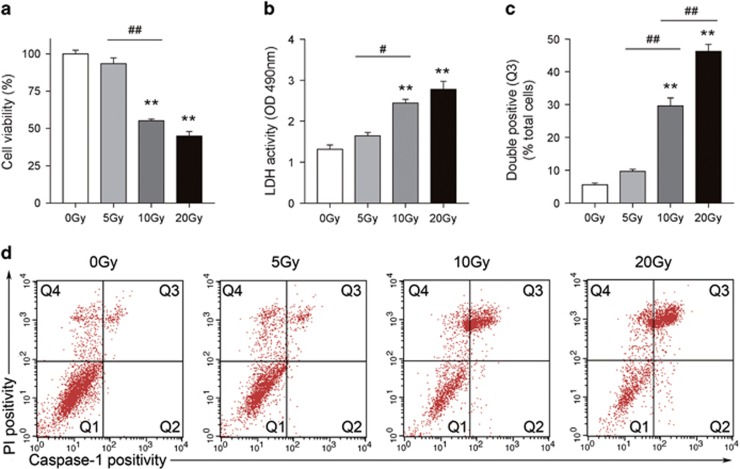
BMDM exposed to radiation shows an increased proportion of pyroptosis. Primarily cultured BMDM were exposed to 5, 10 and 20 Gy radiation, respectively. (**a**) Dose-dependent effects of radiation on the viability of BMDM. (**b**) LDH activity in supernatant was assessed by LDH Cytotoxicity Assay Kit, OD values of 490 nm were present with histogram. (**c**) Detected double positivity of Caspase-1 fluorescent inhibitor probe (FAM-YVAD-FMK) and PI (Q3) using flow cytometry after 24 h. The proportions of Q3 were then presented with histogram. (**d**) Representative flow cytometry scatter plots. Bars represent mean±S.E.M. (*n*=6 or 12). **P<0.001 *versus* 0 Gy group; ^#^*P*<0.01, ^##^*P*<0.001

Cultured BMDM were exposed to a ^60^Co radiation source to attain the desired doses of 5, 10 and 20 Gy. After 24 h, a cell viability assay was performed to evaluate the survival rate of cells. As shown in Figure 1a, groups exposed to radiation (5, 10 and 20 Gy) illustrate a dose-dependent cell loss and reaches low levels by 10 Gy (cell viability 55.27%) and 20 Gy (cell viability 45%, *P*<0.001 *versus* 0 Gy).

Pyroptotic cell death was assessed by measuring the release of LDH and double-positive staining of activated Caspase-1 and propidium iodide (PI).^13^ We detected that radiation (10 and 20 Gy) induced increased activity of LDH dose dependently in BMDM; 10 and 20 Gy radiation induced activity of LDH from 1.318 to 2.442 and 2.782 (OD 490 nm), respectively (*P*<0.001 *versus* 0 Gy, Figure 1b). To determine whether the death of BMDM induced by radiation is a result of pyroptosis, we exposed BMDM to radiation (5, 10, and 20 Gy) and detected activated Caspase-1 and PI using flow cytometry after 24 h. As shown in Figures 1c and d, the proportion of double-positive (activated Caspase-1 and PI) BMDM (Q3) significantly increased in 10 Gy (29.66%) and 20 Gy (46.27%) radiation (*P*<0.001 *versus* 0 Gy), while the 5 Gy group (9.674%) showed no significant difference in comparison with the 0 Gy group (5.604%, *P*>0.05 *versus* 0 Gy).

### Radiation promoted pro-inflammatory cytokine production in bone marrow-derived macrophages

**Figure 2:**
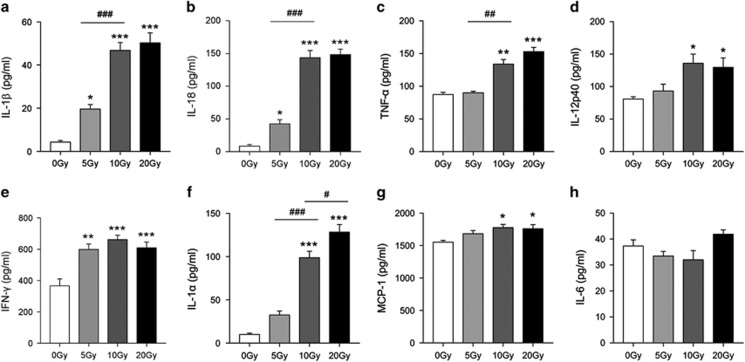
Radiation induces BMDM pro-inflammatory cytokine production in a dose-dependent manner. Primarily cultured BMDM were exposed to 5, 10 and 20 Gy radiation, respectively. After 24 h, ELISA was performed to evaluate the level of pro-inflammatory cytokines in supernatant. Concentration of IL-1*β* (**a**), IL-18 (**b**), TNF-*α* (**c**), IL-12p40 (**d**), IFN-*γ* (**e**), IL-1*α* (**f**), MCP-1 (**g**) and IL-6 (**h**) in supernatant. Bars represent mean±S.E.M. (*n*=6). *^, #^*P*<0.05, **^, ##^*P*<0.01, ***^, ###^*P*<0.001

ELISA analysis of the pro-inflammatory cytokines in supernatant revealed that both 10 and 20 Gy radiation significantly increased the production of IL-1*β*, IL-18, TNF-*α*, IFN-*γ*, IL-1*α*, IL-12p40 and MCP-1 (Figure 2). Notably, 5 Gy radiation also significantly induced the release of IL-1*β*, IL-18 and IFN-*γ* (*P*<0.05, Figures 2a, b and e), but not TNF-*α*, IL-1*α*, IL-12p40 or MCP-1 (*P*>0.05). Nevertheless, radiation stimulation did not have a significant effect on the production of IL-6 (*P*>0.05, Figure 2h). Based on a number of studies of inflammasome activation, we used IL-1*β* instead of both IL-1*β* and IL-18 to test the activation of Caspase-1 in the latter study, as it was deemed more representative and economical.^23, 24^

### Radiation promoted activation of the NLRP3 inflammasome

**Figure 3:**
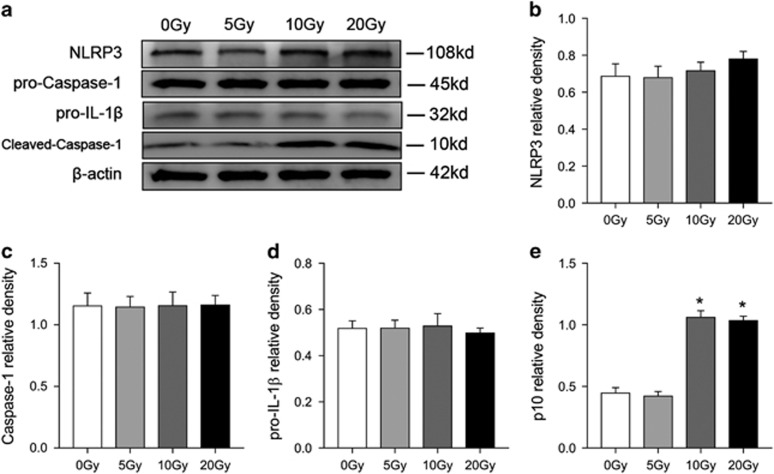
Radiation activates Caspase-1 in BMDM in a dose-dependent pattern. Three hours after radiation, cleared lysates of BMDM were analyzed by Western Blot. (**a**) Immunoblot analysis of NLRP3 inflammasome-related proteins. Relative protein levels of NLRP3 (**b**), Caspase-1 (**c**), pro-IL-1*β* (**d**) and cleaved-Caspase-1 (**e**). Band intensities were then quantified by ImageJ software and the values of the target proteins were normalized to that of *β*-actin. The results are representative of three independent experiments. Bars represent mean±S.E.M. *P<0.001

We performed Western Blot to detect the target protein: NLRP3 (p118), precursors of Caspase-1 (pro-caspase-1, p45), precursors of IL-1*β* (pro-IL-1*β*, p32) and cleaved-Caspase-1 (p10). As shown in Figure 3, 10 and 20 Gy radiation induced Caspase-1 cleavage after 3 h (*p* <0.001 *versus* 0 Gy) while 5 Gy radiation did not show a significant increase of cleaved-Caspase-1 (p10, *P*>0.05 *versus* 0 Gy). Specific comparisons (between treated groups) illustrated no significant difference in cleaved-Caspase-1 (p10) between 10 and 20 Gy radiation groups. However, using RT-PCR, we observed that radiation did not significantly affect the mRNA levels of NLRP3 inflammasome-related gene *Nlrp3, Caspase-1* or *IL-1β* (*P*>0.05 *versus* 0 Gy, Supplementary Figure S1).

As the 10 Gy radiation group shows a significant effect in radiation-induced pyroptosis and Caspase-1 activation, we chose 10 Gy as the treatment dosage in our subsequent research.

### *Nlrp3* knock out suppressed radiation-induced bone marrow-derived macrophages pyroptosis and NLRP3 inflammasome activation

**Figure 4:**
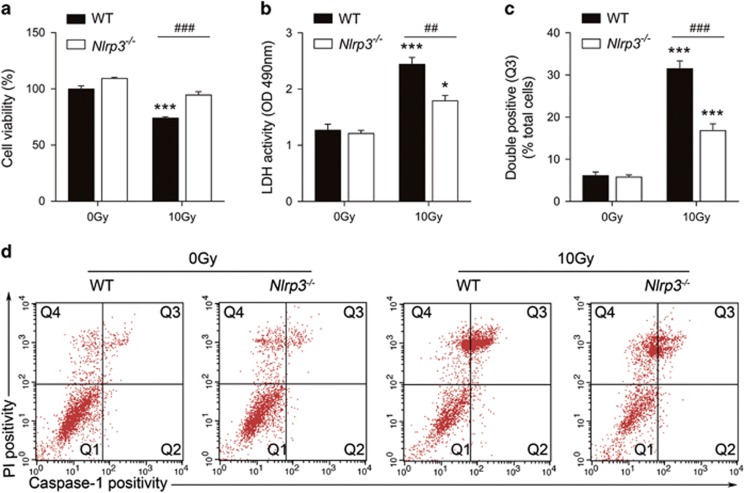
*Nlrp3* knock out inhibited radiation-induced BMDM pyroptosis. BMDM was isolated from *Nlrp3*^*−/−*^ mice and cultured and then exposed to 10 Gy radiation. (**a**) Cell viability was evaluated 24 h after radiation. (**b**) Supernatant LDH activity was assessed and OD values of 490 nm were presented with histogram. (**c**) Detected double positivity of Caspase-1 fluorescent inhibitor probe (FAM-YVAD-FMK) and PI (Q3) using flow cytometry after 24 h. The proportions of Q3 were then presented with histogram. (**d**) Representative flow cytometry scatter plots. Bars represent mean±S.E.M. (*n*=6). *P<0.05, ***P<0.0001 *versus* 0 Gy group; ^##^*P*<0.01, ^###^*P*<0.001

To determine the role of NLRP3 in radiation-induced BMDM pyroptosis, *Nlrp3*^*−/−*^ BMDM was isolated from *Nlrp3*^*−/−*^ mice, cultured and then exposed to10 Gy radiation. Using this strategy, we observed that *Nlrp3*^*−/−*^ BMDM exhibit resistance to radiation-induced cell death (Figure 4a). Knock out of *Nlrp3* rescued 10 Gy induced cell death from 25.98% (wild type (WT)+10 Gy) to 5.45% (*Nlrp3*^*−/−*^+10 Gy, *P*<0.001 *Nlrp3*^*−/−*^+10 Gy *versus* WT+10 Gy). A test of the supernatant LDH showed that knock out of *Nlrp3* suppressed 10 Gy radiation induced LDH release from 2.442 (WT+10 Gy) to 1.792 (*Nlrp3*^*−/−*^+10 Gy, *P*<0.01 *Nlrp3*^*−/−*^+10 Gy *versus* WT+10 Gy, Figure 4b). Furthermore, flow cytometry diagrams indicated that the proportion of pyroptosis induced by radiation was significantly lessened from 31.47% (WT +10 Gy) to 16.83% (*Nlrp3*^*−/−*^+10 Gy, *P*<0.001 *Nlrp3*^*−/−*^+10 Gy *versus* WT+10 Gy, Figures 4c and d).

**Figure 5:**
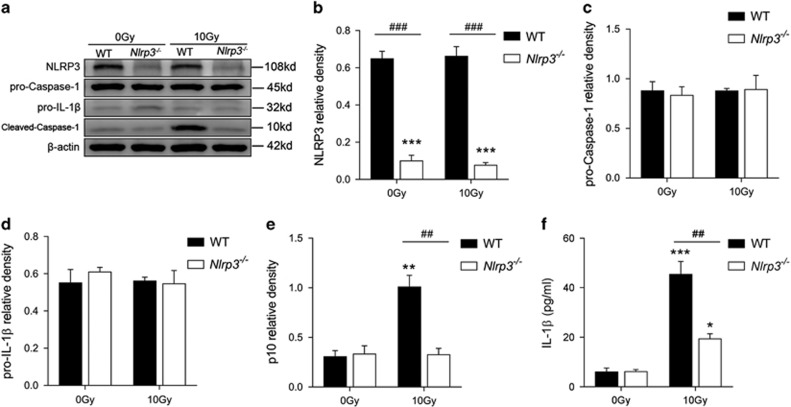
*Nlrp3* knock out inhibited radiation-induced BMDM Caspase-1 activation. (**a**) Western Blot analysis of NLRP3 inflammasome-related proteins. Relative protein levels of NLRP3 (**b**), Caspase-1 (**c**), pro-IL-1*β* (**d**) and cleaved-Caspase-1 (**e**). Band intensities were then quantified by ImageJ software and the values of the target proteins were normalized to that of *β*-actin. The results are representative of three independent experiments. (**f**) Concentration of IL-1*β* in supernatant. Bars represent mean±S.E.M. (*n*=6). *P<0.05, **P<0.01, ***P<0.0001 *versus* 0 Gy WT group; ^##^*P*<0.01, ^###^*P*<0.001

Immunoblotting analysis (Figures 5a–e) revealed that cleaved-Caspase-1 (p10) was elevated in WT BMDM exposed to 10 Gy radiation, while *Nlrp3*^*−/−*^BMDM showed no elevation of cleaved-Caspase-1 (p10), suggesting that knock out of *Nlrp3* can block radiation-induced Caspase-1 activation. Likewise, knock out of *Nlrp3* inhibited radiation-induced IL-1*β* production (*P*<0.001 *Nlrp3*^*−/−*^+10 Gy *versus* WT+10 Gy, Figure 5f).

### *Nlrp3* knock out protected mice from radiation-induced death and Caspase-1 activation

**Figure 6:**
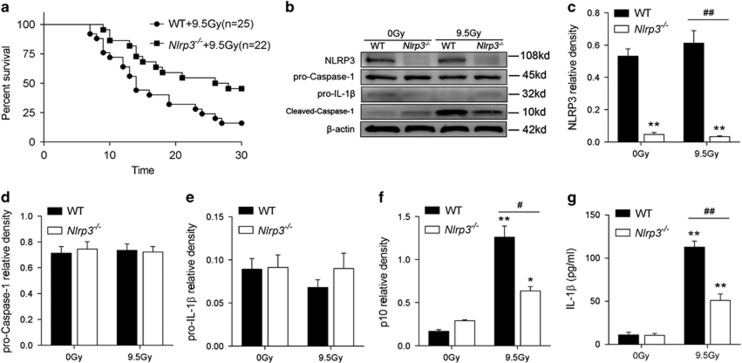
*Nlrp3* knock out protects mice from radiation-induced death and Caspase-1 activation. (**a**) Survival curve: a group of 9.5 Gy radiated *Nlrp3*^*−/−*^ mice (*Nlrp3*^*−/−*^ +9.5 Gy, *n*=22) were monitored for 30 days in comparison with a group of 9.5 Gy radiated WT mice (WT+9.5 Gy, *n*=25), Log-rank (Mantel-Cox) test *P*=0.0175. (**b**–**f**) Western Blot and band intensity analysis of NLRP3-related proteins. The results are representative of three independent experiments. (**g**) ELISA analysis of serum IL-1*β*. Bars represent mean±S.E.M. (*n*=6–8). *P<0.05, **P<0.01 *versus* 0 Gy WT group; ^#^*P*<0.05, ^##^*P*<0.01

To address whether *Nlrp3* knock out can overcome the lethal effect of radiation, a group of 9.5 Gy radiated *Nlrp3*^*−/−*^ mice (*Nlrp3*^*−/−*^ +9.5 Gy, *n*=22) were monitored for 30 days in comparison with a group of 9.5 Gy radiated WT mice (WT+9.5 Gy, *n*=25). *Nlrp3*^*−/−*^ knockout was associated with significantly improved survival at 30 days after 9.5 Gy compared with WT+9.5 Gy group (median survival time: 27 days *versus* 14 days; Figure 6a, log-rank *P*=0.018).

**Figure 7:**
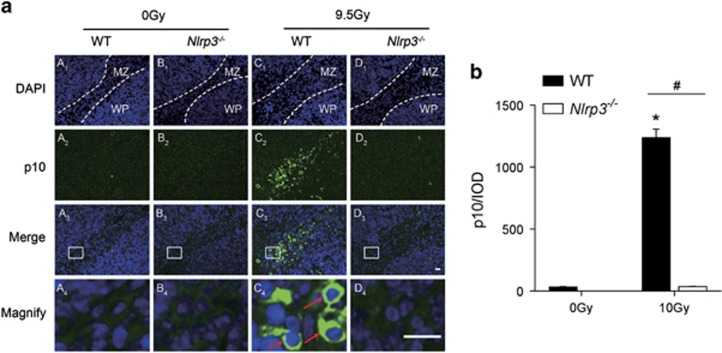
Immunofluorescence staining of DAPI (blue) and cleaved-Caspase-1 (p10, green) in mice spleens 3 h after radiation. (**a**) Representative microscopy images: Red arrows in magnified graph denote the fluorescence of cleaved-Caspase-1 (p10). Dotted lines indicate the border of areas; MZ, marginal zone; WP, white pulp. The results are representative of three independent experiments. Bars indicate scale of 50 *μ*m. (**b**) Cleaved-Caspase-1 (p10) fluorescence was further quantified and presented as integrated optical density (IOD). Bars represent mean±S.E.M. (*n*=6). *P<0.001 *versus* 0 Gy WT group; ^#^*P*<0.001

To further investigate the role of the NLRP3 inflammasome in radiation induced damage, cleaved-Caspase-1 (p10) and IL-1*β* were detected by Western Blot and ELISA, respectively. From Figures 6b–g, we observed that cleaved-Caspase-1 (p10) and IL-1*β* was induced upon radiation stimulation (both *P*<0.001) whereas it was decreased with *Nlrp3* knockout (*P*=0.0015, *P*<0.001, respectively). Similar results were obtained using immunofluorescence analysis (3 h after radiation) for elevated cleaved-Caspase-1 (p10, in green fluorescence) in spleen marginal zone cells (rich in macrophages and follicular dendritic cells) induced by 9.5 Gy radiation. This effect can also be blocked by knocking out *Nlrp3* (Figures 7a and b). Notably, it was difficult to observe staining of cleaved-Caspase-1 in the white pulp cells (rich in lymphocytes) of the spleen.

## Discussion

The present study demonstrates that radiation induces NLRP3 inflammasome activation and pyroptosis in BMDMs. The NLRP3 inflammasome activation, IL-1*β* production, and pyroptosis were downregulated by knockout of *Nlrp3*. These results suggest that radiation activates the NLRP3 inflammasome to induce pyroptosis and IL-1*β* production in macrophages.

Myeloid cells form the first barrier wall against inflammatory stimuli and depletion of these immune cells may damage features of the immune system and may lead to a bystander response and could result in a systemic response syndrome.^6, 25^ However, the death modes and molecular mechanisms of immune cells exposed to radiation are only beginning to be appreciated and need further evidence to be understood. Using primary cultured BMDMs, our study demonstrated that radiation induces macrophage death and pro-inflammatory cytokine (includes M1 cytokines such as TNF-α, IFN-*γ* and IL-12) release in a dose-dependent manner (Figures 1 and 2), which is broadly consistent with previous studies.^5, 9, 10^ We found a significant increase in MCP-1 in radiation treated groups, which was also shown by DeBo *et al.*, although not in Stoecklein's or Siva's research.^26, 27^ Additionally, we did not detect obvious increases in IL-6 after radiation, which is not consistent with several previous studies.^28^ These differences in findings may be because of the use of different cell types and/or radiation sources.

Pyroptosis, defined as Caspase-1-dependent programmed and pro-inflammatory cell death, is distinct from any other programmed cell death and results in cell lysis and pro-inflammatory cytokine release.^29^ Pyroptosis is triggered by various pathological stimuli, such as stroke, heart attack, cancer, microbial or liver inflammation and fibrosis.^30, 31^ Unlike Caspase-3-dependent apoptosis, pyroptosis is typically mediated by Caspase-1 activation following inflammasome complex formation.^16^ Another apparent feature of pyroptosis is pore formation, which permits cytosolic contents such as LDH and pro-inflammatory cytokine release and fluorochrome-conjugated annexin V, 7-aminoactinomycin (7-AAD) or PI enter the cell.^13^ Nevertheless, membrane impermeant dyes such as 7-AAD or PI do not stain apoptotic cells.^13^ Although Stoecklein *et al.* discussed the role of radiation-induced immune cell pyroptosis in their good work, there remains some confusion about the methods of pyroptosis detection.^5^ Stoecklein *et al.* first stained cells with annexin V and cleaved-Caspase-1 (p10) and found annexin V^+^ cells express higher cleaved-Caspase-1 (p10) than annexin V^−^ cells after radiation. Then, they stained another group of radiated cells with annexin V and PI and found that radiation increased double-positive cells (annexin V^+^/PI^+^). Taken together, they concluded that these annexin V^+^/PI^+^ cells may also express more cleaved-Caspase-1 (p10) and be more subject to pyroptotic death. However, in an indirect way, the method cannot exclude annexin V^+^/PI^+^/p10^−^ cells from annexin V^+^/PI^+^ cells, and these type of cells (annexin V^+^/PI^+^/p10^−^) are known as apoptotic cells in the late stage, not pyroptotic cells. Indeed, although controversial, many studies have used relatively direct and exact methods to detect pyroptosis such as double staining cells with FAM-YVAD-FMK (a marker of activated Caspase-1) and PI or SYTOX Blue DNA intercalation staining (a marker of pore formation in the plasma membrane).^30, 32^ Detection of LDH release (as a marker of pore formation in the plasma membrane) is also used widely to identify pyroptosis.^32^ Some labs like Jiahuai *et al.*'s and Lei *et al.*'s also have made great efforts to explore the morphologic characteristics of pyroptosis by electron microscopy and have made certain achievements recently that may contribute to the future detection of pyroptosis.^30, 33^ In this study, detection of active caspase 1 and PI double stain were used in combination with an LDH release assay to detect and quantify pyroptosis. Our current data confirmed that radiation induces BMDM pyroptosis in a dose-dependent manner. Importantly, the 5 Gy radiation group did not show a significant increase of double-positive Caspase-1 and PI, nor did they show an elevation of LDH activity compared with the control group (0 Gy), which suggests a BMDM tolerance to relatively low doses of radiation (Figure 1). Many studies have shown an active role for macrophages under relatively low doses of radiation (2 Gy or 8 Gy) when many other cell types exhibit a marked loss.^34, 35, 36^ Based on these results and previous research, we favor the hypothesis that low dose radiation activates the macrophages that then mediate a variety of biological effects while relative high dose radiation kills the macrophages in a pyroptotic manner and mediates various types of severe damage.

As pyroptosis occurs after activation of caspase-1 and many studies that have focused on the inflammatory responses under radiation detected controversial results about the links between radiation exposure and NLRP3 inflammasome activation, we then tested the role of the NLRP3 inflammasome in radiation and in the pyroptosis induced by radiation in cultured BMDM using RT-PCR, immunoblotting and ELISA techniques. Interestingly, the results imply that BMDM exposed to radiation shows evidence of dose-dependent Caspase-1 activation and IL-1*β* production (Figures 2 and 3) and that the Caspase-1 activation and IL-1*β* production is dependent on NLRP3, since *Nlrp3* knock out diminishes the quantity of cleaved-Caspase-1 (p10) and IL-1*β* (Figures 4 and 5). This conclusion was confirmed by *in vivo* research (Figures 6 and 7). More vitally, *Nlrp3* knock out can also significantly lessen the proportion of pyroptosis induced by radiation. Accordingly, we believe that radiation can induce pyroptosis through activating the NLRP3 inflammasome. This finding is novel and further supports the idea that NLRP3-Caspase-1 inflammasome activation is essential in radiation induced cell and tissue damage.^19, 21^ Whereas, we noticed that depletion of NLRP3 rescued only a fraction of BMDM from radiation induced death and pyroptosis and Caspase-1 activation, which supports the hypothesis that radiation-induced Caspase-1 activation and pyroptosis is not wholly NLRP3-dependent. As radiation can break gut mucosa and lung epithelial cells which, in turn, increase susceptibility to infections, other inflammasomes such as NLRP1, NLRC4 or AIM2 that can recognize PAMPs may also contribute to radiation-induced caspase-1 activation.^21, 37, 38^ Stoecklein found that *Nlrp3*^*−/−*^ mice did not show a protective effect against radiation-induced immune cell loss. This could be partially explained by the fact that relative low dose radiation induces immune cell loss indirectly, such as through PAMPs activating other inflammasomes, which further supports our previous inference. It is also worth noting that, we did not observe a significant change in the expression of NLRP3 inflammasome-related protein Nlrp3, Caspase-1 and IL-1*β* 3 h after radiation (Supplementary Figure S1; Figure 3), while other studies had detected elevated expression levels of the NLRP3 inflammasome-related protein after at least 1 day.^18, 19, 20^ The activation of the NLRP3 inflammasome is generally believed to require two signals, signal 1 being Toll-like receptor activation leading to cellular priming and upregulation of NLRP3 expression and signal 2 being an additional stimulation of these cells with PAMPs or DAMPs.^39^ As we know, protein expression is time-dependent and those studies that detected elevated NLRP3 inflammasome related protein at least 24 h after radiation cannot avoid the effect of radiation-induced cytokines on NLRP3 inflammasome-related protein expression. Indeed, BMDM constitutively express NLRP3 which make it possible for agonists to directly activate NLRP3 inflammasome.^40^ The direct effects of radiation, such as damage to lysosomes, nuclear DNA or mitochondrial DNA, could be the signal 2 of NLRP3 inflammasome activation.^41^ In addition, different tissue or cell types may react differently to iron radiation.^42^ Based on these findings, we assume that radiation does not directly affect the expression of NLRP3 inflammasome-related protein in the early stage.

To the best of our knowledge, we reported here for the first time that NLRP3 inflammasome activation mediates radiation-induced pyroptosis in BMDMs. We further assume that radiation can directly activate the NLRP3 inflammasome without affecting the expression level and indirectly activate other inflammasomes in the later period. Targeting the NLRP3 inflammasome and secondary pyroptosis may represent a novel strategy to limit the radiation-induced loss of immune cells, cascades of pro-inflammatory cytokines and related tissue damage. However, there are still limitations that need further exploration. For example, the mechanism of radiation activation of the NLRP3 inflammasome, the downstream cytokine induction of Caspase-1 activation and the role of other inflammasomes in radiation induced cell or tissue damage are not clear.

## Materials and Methods

### Animals

WT C57BL/6J male mice, 5-7 weeks age, were purchased from Super-B&K Laboratory Animal Corp. Ltd. (Shanghai, China). Male *Nlrp3*^−/−^ mice on the C57BL/6J genetic background were from Model Animal Research Centre (AAALAC accredited, Nanjing University, China). Housed in specific-pathogen-free animal facility for at least 5 days under a 12-h light/dark cycle, the animals were given access to water and standard laboratory chow *ad libitum*. Those knockout mice and cells were identified by PCR (sequences of primers were presented in Supplementary Table S2), DNA sequencing, immunoblotting and immunofluorescence staining (Supplementary Figure S2). All animal procedures used in this study were approved by the Institutional Animal Care and Use Committee of the Second Military Medical University (No. 20120025, Shanghai, China).

### Cells isolation and culture

The isolation and culture of BMDM was performed as Pineda-Torra *et al.* described.^43^ At first, briefly, animals were killed by cervical dislocation and soaked in 75% ethanol. Then, femurs and tibias were harvested and the bone marrow cells from all bones were flushed out. After centrifuging for 5 min at 310 × *g*, Erythrocytes were eliminated using Red Blood Cell Lysing Buffer (Sigma-Aldrich, St. Louis, MO, USAhttp://www.sigmaaldrich.com).The remaining cells were seeded in plates and incubated in complete medium with 50 mg/ml recombinant mouse M-CSF (R&D Systems, Inc., Minneapolis, MN, USA) for 7 days to form proliferative nonactivated cells (also named M0 macrophages).

### *γ*-ray radiation

*In vivo*, mice were exposed to whole-body radiation by timed exposure to ^60^Co radiation source (Faculty of Naval Medicine, Second Military Medical University) with a dose rate of 1.63 Gy/min and cumulative radiation dosage of 9.50 Gy. For *in vitro* assay, BMDM were grown as a monolayer and were exposed to ^60^Co radiation source to attain the desired dose of 5, 10 and 20 Gy at a dose rate of 1.80 Gy/min.

### Cell viability assay

Thirty thousand cells per well were seeded into 96-well plates in 100 *μ*l of RPMI 1640 supplemented with 10% FBS, 0.1 mg/ml Penicillin/Streptomycin (P/S) and 50 mg/ml recombinant mouse M-CSF and incubated for 7 days as described before. Then, the cells were exposed to ^60^Co radiation and the number of surviving cells was measured by Cell Counting Kit-8 (CCK-8) (Dojindo Laboratories, Kumamoto, Japan) after 24 h. Data acquisition was performed on DENLEY DRAGON Wellscan MK 3 (Thermo, Vantaa, Finland). Cell viability was calculated according to the formula: cell viability (%)=[(As−Ab)/(Ac−Ab) × 100%, where As, Ac and Ab represent the A450 in treated, untreated and blank groups, respectively.

### Determination of BMDM pyroptosis

Pyroptotic cell death was assessed by measuring the release of LDH and double-positive staining of activated Caspase-1 and PI in BMDM. Released LDH in the cell culture supernatant was detected using LDH Cytotoxicity Assay Kit (Beyotime, Haimen, Jiangsu, China) and the activated Caspase-1 and PI were detected by FAM-FLICA Caspase-1 Assay Kit (ImmunoChemistry Technologies, LCC, Bloomington, MN, USA) according to the manufacturer's instruction. Stained cells were then analyzed by flow cytometry (BD FACSCalibur, Becton, Dickinson and Company, Franklin Lakes, NJ, USA).

### Quantitative real-time polymerase chain reaction (qRT-PCR)

The total RNA was isolated from adhered BMDM (3 h after radiation) using Trizol reagent (Life Technologies, Carlsbad, CA, USA). The reverse transcript (cDNA) was synthesized from 1 *μ*g of total RNA and PrimeScript RT Master Mix (Takara Biotechnology, Dalian, China). QRT-PCR was performed with 2 *μ*l first-strand cDNA solution in combination with a Fast start Universal Probe Master Mix, in a final volume of 20 *μ*l. The primers used in this study are listed in Supplementary Table S1. All samples were run in triplicate and underwent 40 amplification cycles on an Applied Biosystems 7500 Real-Time PCR System (Life Technologies Corporation, USA) following the manufacturer's protocols. Expression values of NLRP3, Caspase-1 and pro-IL-1*β* were normalized to the value of the endogenous standard GAPDH and calculated by using the comparative cycle threshold (ΔΔC_t_) method.^44^

### Immunoblot analysis

For Western Blot analysis, adhered BMDM (3 h after radiation) or spleens of mice (3 h after radiation) were lysed and the protein concentrations were measured as we described previously.^45^ Cleared lysates were separated by 10% SDS-PAGE, transferred onto NC membranes and then blocked for 2 h at room temperature with 5% nonfat dried milk. NLRP3, pro-Caspase-1 (p45) and cleaved-Caspase-1 (p10) detection was accomplished by probing the membranes with anti-NLRP3 Ab (AdipoGen Corp., San Diego, CA, USA), anti-Caspase-1 (p45) Ab (Abcam, Cambridge, UK) and anti-cleaved-Caspase-1 (p10) Ab (Santa Cruz Biotechnology, lnc., Dallas, TX, USA), and exposed with an Amersham Imager 600 (GE Healthcare Bio-Sciences AB, Uppsala, Sweden). The membranes were then stripped, reprobed with anti-*β*-actin and exposed again to detect the endogenous standard *β*-actin. ImageJ software was then used to scan and quantify the immunoblots. The band intensity values of the target proteins were normalized to that of *β*-actin.

### Cytokine measurement

Cell-free culture supernatants were tested for IL-1*β*,IL-18 IL-1*α*, TNF-*α*, IFN-*γ*, IL-6, IL-12p40 and MCP-1 concentrations using ELISA kits (Dakewe Biotech Company Ltd., Shenzhen, China) following the manufacturer's protocols. Data acquisition was performed on DENLEY DRAGON Wellscan MK 3 (Thermo, Finland).

### Immunofluorescence staining

After anesthetizing, the mice were transcardially perfused with normal saline (0.9%) and spleen tissues were isolated and fixed in fresh paraformaldehyde solution (4%, PH 7.4). Horizontal slices were prepared and blocked with 1% bovine serum albumin (BSA; Sigma-Aldrich) in PBS containing 0.05% Tween 20 (0.05% PBS-T) for 2 h. Specimens were then incubated with primary described anti-cleaved-Caspase-1 (p10) Ab overnight at 4 °C and Alexa Fluor 488 (green) labeled donkey anti-rabbit IgG were then loaded for 2 h at room temperature in the dark. After washing, DAPI (4′,6-diamidino-2-phenylindole) was used for nuclear staining. Stained specimens were visualized with an Olympus Research Inverted System Microscope IX71 (Olympus, Tokyo, Japan). Fluorescence intensity of cleaved-Caspase-1 (p10) was quantified by Image Pro Plus 6.0 software (Media Cybernetics, Silver Spring, MD, USA).

### Statistical analysis

The data are expressed as the mean±S.E.M. and analyzed for statistical significance using GraphPad Prism 5.0.1 (GraphPad Software, La Jolla, CA, USA). One-way ANOVA was used to detected statistical significance among group means and Bonferroni *post-hoc* analysis was used to compare specific groups when ANOVA showed significant differences. *P*<0.05 was considered to be statistically significant.

## References

[bib1] Wilkins A, Mossop H, Syndikus I, Khoo V, Bloomfield D, Parker C et al. Hypofractionated radiotherapy versus conventionally fractionated radiotherapy for patients with intermediate-risk localised prostate cancer: 2-year patient-reported outcomes of the randomised, non-inferiority, phase 3 CHHiP trial. Lancet Oncol 2015; 16: 1605–1616. 26522334 10.1016/S1470-2045(15)00280-6 PMC4664817

[bib2] Bradley JD, Paulus R, Komaki R, Masters G, Blumenschein G, Schild S et al. Standard-dose versus high-dose conformal radiotherapy with concurrent and consolidation carboplatin plus paclitaxel with or without cetuximab for patients with stage IIIA or IIIB non-small-cell lung cancer (RTOG 0617): a randomised, two-by-two factorial phase 3 study. Lancet Oncol 2015; 16: 187–199. 25601342 10.1016/S1470-2045(14)71207-0 PMC4419359

[bib3] De Meerleer G, Khoo V, Escudier B, Joniau S, Bossi A, Ost P et al. Radiotherapy for renal-cell carcinoma. Lancet Oncol 2014; 15: e170–e177. 24694640 10.1016/S1470-2045(13)70569-2

[bib4] Kim JH, Jenrow KA, Brown SL. Mechanisms of radiation-induced normal tissue toxicity and implications for future clinical trials. Radiat oncol j 2014; 32: 103–115. 25324981 10.3857/roj.2014.32.3.103 PMC4194292

[bib5] Stoecklein VM, Osuka A, Ishikawa S, Lederer MR, Wanke-Jellinek L, Lederer JA. Radiation exposure induces inflammasome pathway activation in immune cells. J immunol 2015; 194: 1178–1189. 25539818 10.4049/jimmunol.1303051 PMC4326002

[bib6] Ratikan JA, Micewicz ED, Xie MW, Schaue D. Radiation takes its toll. Cancer lett 2015; 368: 238–245. 25819030 10.1016/j.canlet.2015.03.031 PMC4578968

[bib7] Ahn GO, Tseng D, Liao CH, Dorie MJ, Czechowicz A, Brown JM. Inhibition of Mac-1 (CD11b/CD18) enhances tumor response to radiation by reducing myeloid cell recruitment. Proc Natl Acad Sci USA 2010; 107: 8363–8368. 20404138 10.1073/pnas.0911378107 PMC2889597

[bib8] Cappuccini F, Eldh T, Bruder D, Gereke M, Jastrow H, Schulze-Osthoff K et al. New insights into the molecular pathology of radiation-induced pneumopathy. Radiother oncol 2011; 101: 86–92. 21722981 10.1016/j.radonc.2011.05.064

[bib9] Farooque A, Afrin F, Adhikari JS, Dwarakanath BS. Polarization of macrophages towards M1 phenotype by a combination of 2-deoxy-d-glucose and radiation: implications for tumor therapy. Immunobiology 2016; 221: 269–281. 26597503 10.1016/j.imbio.2015.10.009

[bib10] Klug F, Prakash H, Huber PE, Seibel T, Bender N, Halama N et al. Low-dose irradiation programs macrophage differentiation to an iNOS(+)/M1 phenotype that orchestrates effective T cell immunotherapy. Cancer cell 2013; 24: 589–602. 24209604 10.1016/j.ccr.2013.09.014

[bib11] Malhotra P, Adhikari M, Mishra S, Singh S, Kumar P, Singh SK et al. N-acetyl tryptophan glucopyranoside (NATG) as a countermeasure against gamma radiation-induced immunosuppression in murine macrophage J774A.1 cells. Free rad res 2016; 50: 1–14. 10.1080/10715762.2016.1235788 27620851

[bib12] Abe J, Morrell C. Pyroptosis as a regulated form of necrosis: PI+/annexin V−/high caspase 1/low caspase 9 activity in cells=pyroptosis? Circ res 2016; 118: 1457–1460. 27174943 10.1161/CIRCRESAHA.116.308699 PMC5137941

[bib13] Miao EA, Rajan JV, Aderem A. Caspase-1-induced pyroptotic cell death. Immunol rev 2011; 243: 206–214. 21884178 10.1111/j.1600-065X.2011.01044.x PMC3609431

[bib14] Croker BA, O'Donnell JA, Gerlic M. Pyroptotic death storms and cytopenia. Curr opin immunol 2014; 26: 128–137. 24556409 10.1016/j.coi.2013.12.002

[bib15] Guo H, Callaway JB, Ting JP. Inflammasomes: mechanism of action, role in disease, and therapeutics. Nature med 2015; 21: 677–687. 26121197 10.1038/nm.3893 PMC4519035

[bib16] Aachoui Y, Sagulenko V, Miao EA, Stacey KJ. Inflammasome-mediated pyroptotic and apoptotic cell death, and defense against infection. Curr opin microbiol 2013; 16: 319–326. 23707339 10.1016/j.mib.2013.04.004 PMC3742712

[bib17] Brennan MA, Cookson BT. Salmonella induces macrophage death by caspase-1-dependent necrosis. Mol microbiol 2000; 38: 31–40. 11029688 10.1046/j.1365-2958.2000.02103.x

[bib18] Sohn SH, Lee JM, Park S, Yoo H, Kang JW, Shin D et al. The inflammasome accelerates radiation-induced lung inflammation and fibrosis in mice. Environ toxicol pharmacol 2015; 39: 917–926. 25805627 10.1016/j.etap.2015.02.019

[bib19] Ortiz F, Acuna-Castroviejo D, Doerrier C, Dayoub JC, Lopez LC, Venegas C et al. Melatonin blunts the mitochondrial/NLRP3 connection and protects against radiation-induced oral mucositis. J pineal res 2015; 58: 34–49. 25388914 10.1111/jpi.12191

[bib20] Shin D, Lee G, Sohn SH, Park S, Jung KH, Lee JM et al. Regulatory T cells contribute to the inhibition of radiation-induced acute lung inflammation via bee venom phospholipase A(2) in mice. Toxins 2016; 8: 131. 10.3390/toxins8050131 PMC4885046 27144583

[bib21] Feldmeyer L, Keller M, Niklaus G, Hohl D, Werner S, Beer HD. The inflammasome mediates UVB-induced activation and secretion of interleukin-1beta by keratinocytes. Curr biol 2007; 17: 1140–1145. 17600714 10.1016/j.cub.2007.05.074

[bib22] Li T, Li L, Li F, Liu Y. X-ray irradiation accelerates senescence in hippocampal neural stem/progenitor cells via caspase-1 activation. Neurosci lett 2015; 585: 60–65. 25445379 10.1016/j.neulet.2014.11.028

[bib23] Sheedy FJ, Grebe A, Rayner KJ, Kalantari P, Ramkhelawon B, Carpenter SB et al. CD36 coordinates NLRP3 inflammasome activation by facilitating intracellular nucleation of soluble ligands into particulate ligands in sterile inflammation. Nature immunol 2013; 14: 812–820. 23812099 10.1038/ni.2639 PMC3720827

[bib24] Huai W, Zhao R, Song H, Zhao J, Zhang L, Zhang L et al. Aryl hydrocarbon receptor negatively regulates NLRP3 inflammasome activity by inhibiting NLRP3 transcription. Nature commun 2014; 5: 4738. 25141024 10.1038/ncomms5738

[bib25] Prise KM, Schettino G, Folkard M, Held KD. New insights on cell death from radiation exposure. Lancet Oncol 2005; 6: 520–528. 15992701 10.1016/S1470-2045(05)70246-1

[bib26] DeBo RJ, Lees CJ, Dugan GO, Caudell DL, Michalson KT, Hanbury DB et al. Late effects of total-body gamma irradiation on cardiac structure and function in male rhesus macaques. Radiat res 2016; 186: 55–64. 27333082 10.1667/RR14357.1 PMC5068576

[bib27] Siva S, MacManus M, Kron T, Best N, Smith J, Lobachevsky P et al. A pattern of early radiation-induced inflammatory cytokine expression is associated with lung toxicity in patients with non-small cell lung cancer. PloS one 2014; 9: e109560. 25289758 10.1371/journal.pone.0109560 PMC4188745

[bib28] Oh ET, Park MT, Song MJ, Lee H, Cho YU, Kim SJ et al. Radiation-induced angiogenic signaling pathway in endothelial cells obtained from normal and cancer tissue of human breast. Oncogene 2014; 33: 1229–1238. 23503466 10.1038/onc.2013.70

[bib29] LaRock CN, Cookson BT. Burning down the house: cellular actions during pyroptosis. PLoS pathog 2013; 9: e1003793. 24367258 10.1371/journal.ppat.1003793 PMC3868505

[bib30] Geng Y, Ma Q, Liu YN, Peng N, Yuan FF, Li XG et al. Heatstroke induces liver injury via IL-1beta and HMGB1-induced pyroptosis. J hepatol 2015; 63: 622–633. 25931416 10.1016/j.jhep.2015.04.010

[bib31] Wree A, Eguchi A, McGeough MD, Pena CA, Johnson CD, Canbay A et al. NLRP3 inflammasome activation results in hepatocyte pyroptosis, liver inflammation, and fibrosis in mice. Hepatology (Baltimore, Md) 2014; 59: 898–910. 10.1002/hep.26592 PMC4008151 23813842

[bib32] Chen H, Lu Y, Cao Z, Ma Q, Pi H, Fang Y et al. Cadmium induces NLRP3 inflammasome-dependent pyroptosis in vascular endothelial cells. Toxicol lett 2016; 246: 7–16. 26809137 10.1016/j.toxlet.2016.01.014

[bib33] Chen X, He WT, Hu L, Li J, Fang Y, Wang X et al. Pyroptosis is driven by non-selective gasdermin-D pore and its morphology is different from MLKL channel-mediated necroptosis. Cell res 2016; 26: 1007–1020. 27573174 10.1038/cr.2016.100 PMC5034106

[bib34] De Palma M, Coukos G, Hanahan D. A new twist on radiation oncology: low-dose irradiation elicits immunostimulatory macrophages that unlock barriers to tumor immunotherapy. Cancer cell 2013; 24: 559–561. 24229704 10.1016/j.ccr.2013.10.019

[bib35] Seifert L, Werba G, Tiwari S, Giao Ly NN, Nguy S, Alothman S et al. Radiation therapy induces macrophages to suppress T-cell responses against pancreatic tumors in mice. Gastroenterology 2016; 150: 1659–1672.e1655. 26946344 10.1053/j.gastro.2016.02.070 PMC4909514

[bib36] Mukherjee D, Coates PJ, Lorimore SA, Wright EG. Responses to ionizing radiation mediated by inflammatory mechanisms. J pathol 2014; 232: 289–299. 24254983 10.1002/path.4299

[bib37] Coggle JE, Lambert BE, Moores SR. Radiation effects in the lung. Environ health perspect 1986; 70: 261–291. 3549278 10.1289/ehp.8670261 PMC1474274

[bib38] Francois A, Milliat F, Guipaud O, Benderitter M. Inflammation and immunity in radiation damage to the gut mucosa. BioMed res int 2013; 2013: 123241. 23586015 10.1155/2013/123241 PMC3614034

[bib39] Anand PK, Malireddi RK, Kanneganti TD. Role of the nlrp3 inflammasome in microbial infection. Front microbiol 2011; 2: 12. 21687408 10.3389/fmicb.2011.00012 PMC3109351

[bib40] Chen M, Wang H, Chen W, Meng G. Regulation of adaptive immunity by the NLRP3 inflammasome. Int immunopharmacol 2011; 11: 549–554. 21118671 10.1016/j.intimp.2010.11.025

[bib41] Sutterwala FS, Haasken S, Cassel SL. Mechanism of NLRP3 inflammasome activation. Ann NY Acad Sci 2014; 1319: 82–95. 24840700 10.1111/nyas.12458 PMC4074217

[bib42] Vianale G, Reale M, Amerio P, Stefanachi M, Di Luzio S, Muraro R. Extremely low frequency electromagnetic field enhances human keratinocyte cell growth and decreases proinflammatory chemokine production. Br j dermatol 2008; 158: 1189–1196. 18410412 10.1111/j.1365-2133.2008.08540.x

[bib43] Pineda-Torra I, Gage M, de Juan A, Pello OM. Isolation, culture, and polarization of murine bone marrow-derived and peritoneal macrophages. Meth mol biol 2015; 1339: 101–109. 10.1007/978-1-4939-2929-0_6 26445783

[bib44] Lebeaupin C, Proics E, de Bieville CH, Rousseau D, Bonnafous S, Patouraux S et al. ER stress induces NLRP3 inflammasome activation and hepatocyte death. Cell death dis 2015; 6: e1879. 26355342 10.1038/cddis.2015.248 PMC4650444

[bib45] Zhang ZT, Du XM, Ma XJ, Zong Y, Chen JK, Yu CL et al. Activation of the NLRP3 inflammasome in lipopolysaccharide-induced mouse fatigue and its relevance to chronic fatigue syndrome. J neuroinflammation 2016; 13: 71. 27048470 10.1186/s12974-016-0539-1 PMC4822300

