# Supplementary material for: NLRP3 inflammasome activation mediates radiation-induced pyroptosis in bone marrow-derived macrophages
Source: Cell Death Dis. 2017 Feb 2;8(2):e2579–. doi: 10.1038/cddis.2016.460 (PMC5386456; doi:10.1038/cddis.2016.460)
Supplement: Supplementary Information [file cddis2016460x1.docx]

**NLRP3 Inflammasome Activation Mediates Radiation-Induced Pyroptosis in bone marrow-derived macrophages**

Yan-gang Liu^1,4^, Ji-kuai Chen^1,4^, Zi-teng Zhang^1,4^, Xiu-juan Ma^1^, Yong-chun Chen^1^, Xiu-ming Du^1^, Hong Liu^3^, Ying Zong*^,1^, Guo-cai Lu*^,1,2^

^1^ Department of Health Toxicology，College of Tropical Medicine and Public Health，Second Military Medical University, Shanghai 200433, China

^2^ CTI Biotechnology (Suzhou) Co., Ltd, Jiangsu 215300, China

^3^ Radiology Department X-ray Room, The Second People’s Hospital, Neijiang, Sichuan 641100, China

^4^ These authors contributed equally to this work.

^*^ Correspondence: Prof. Guo-cai Lu, Department of Health Toxicology, College of Tropical Medicine and Public Health，Second Military Medical University, NO 800，Xiang-yin Road, Shanghai 200433, China; CTI Biotechnology (Suzhou) Co., Ltd, Jiangsu 215300, China. Tel: +86 51236802386. E-mail: newdrug@smmu.edu.cn. Prof. Ying Zong, Department of Health Toxicology, College of Tropical Medicine and Public Health, Second Military Medical University, NO 800, Xiang-yin Road, Shanghai 200433, China. Tel: +86 2181871035. E-mail: standyup@hotmail.com.

**Supplementary Table: primers used in this study**

Table S1 Sequences of primers used for RT-PCR

| Names | Forward primer(5’-3’) | Reverse primer(3’-5’) |
| --- | --- | --- |
| NLRP3 | ACCAGCCAGAGTGGAATGAC | ATGGAGATGCGGGAGAGATA |
| Pro-Caspase-1 | TGGTCTTGTGACTTGGAGGA | TATTGGCACGATTCTCAGCA |
| Pro-IL-1β | CTCACAAGCAGAGCACAAGC | TCCAGCCCATACTTTAGGAAGA |

Table S2 Sequences of primers used for genomic PCR

| Names | Sequences | Direction |
| --- | --- | --- |
| *Nlrp3*- e1-sens1 | CCTCTTTATATGCACACAATTCCAC | 5’-3’ |
| *Nlrp3-* e4-sens1 | GCAAGCTTCATGATAATGTGACTG | 5’-3’ |
| *Nlrp3-*-e4-anti2 | GGCCAGGCTGACAGACCCTCAT | 3’-5’ |

**Supplementary Figures**


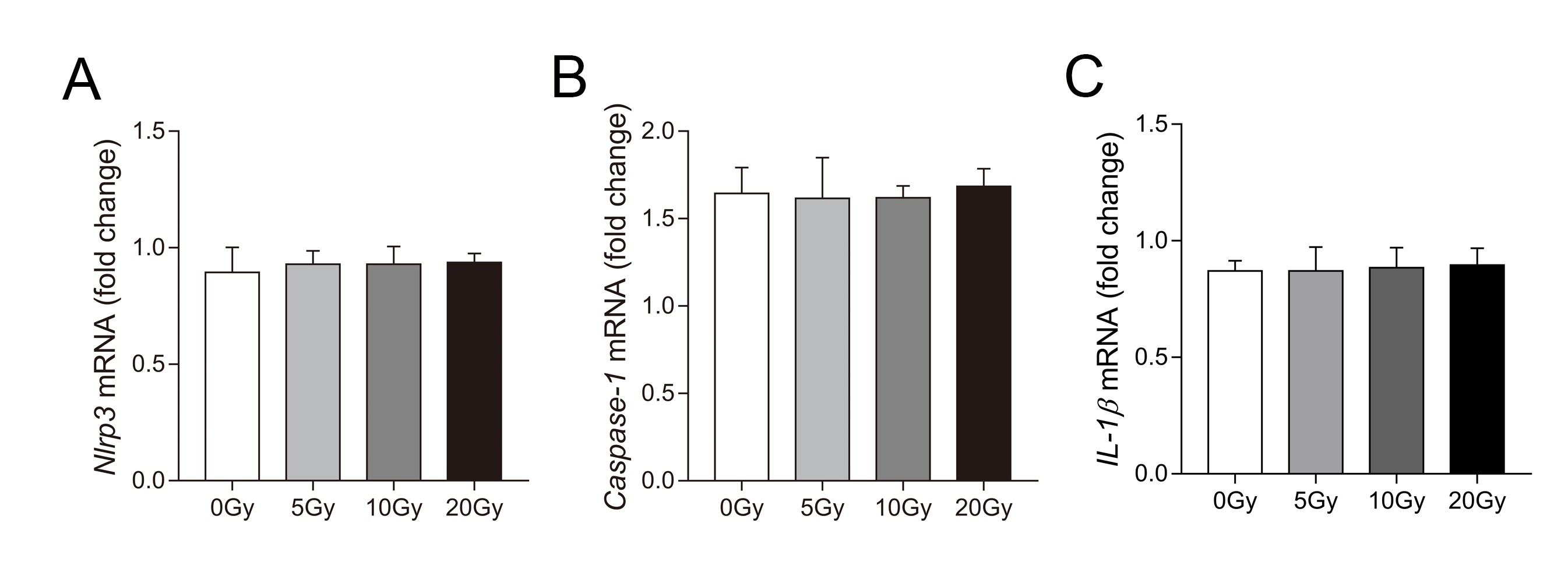


Figure S1 NLRP3 inflammasome related gene expression in BMDM. Cultured BMDM were exposed to 5 Gy, 10 Gy and 20 Gy radiation, respectively. RT-PCR was performed to analyze the mRNA level of *Nlrp3* (**A**)*, Caspase-1* (**B**)*, IL-1β* (**C**). Data were expressed as relative fold change (n=6).


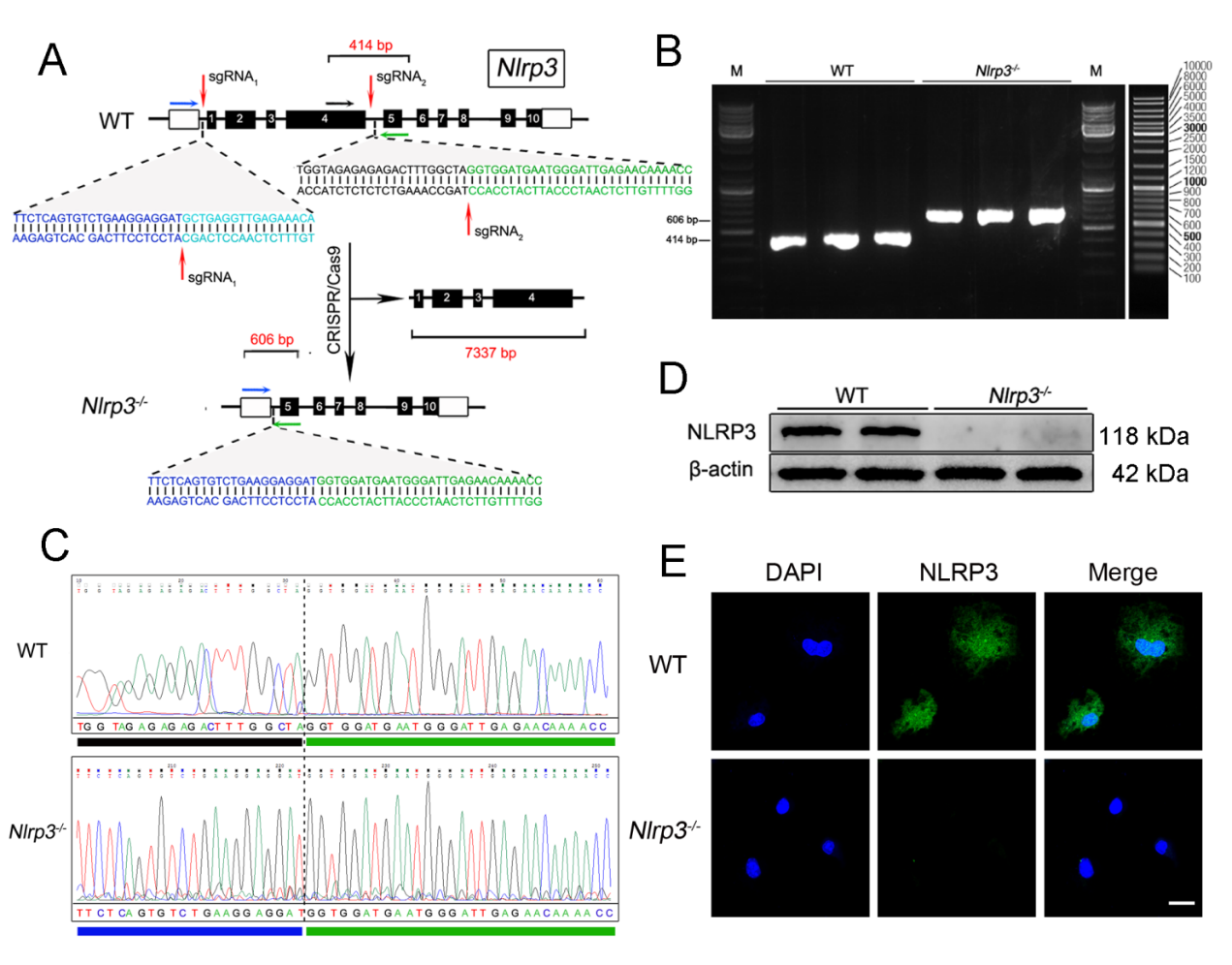


Figure S2 Identification of *Nlrp3*^-/-^ mice and cells. DNA samples were extracted from the muscle biopsies of Wide-type (WT) and Nlrp3^-/-^ mice and identified by PCR and sequencing. To verify the results in mice, the level of NLRP3 protein in cultured WT and *Nlrp3^-/-^* BMDM were detected by Western Blot analysis and immunofluorescence staining. (**A**) Schematic depiction of the paired-KO strategy for *Nlrp3* knockout. Vertical red arrows indicate targeting sites for sgRNA. The positions of the designed primers for genomic PCR are shown as horizontal arrows (blue arrows indicate *Nlrp3*- e1-sens1, black arrow indicates *Nlrp3*-e4-sens1 and green arrows indicate *Nlrp3-*e4-anti2, sequence see Table S2). Expected PCR products for the WT mice are 414 bp (between black and green arrow) and for the *Nlrp3^-/-^* mice are 606 bp (between blue and green arrow). Numbers in black board represent the order of exons within *Nlrp3*. (**B**) Representative agarose gel picture indicates that PCR products from WT mice are 414 bp events and *Nlrp3^-/-^* mice are 606 bp events. M, size marker. (**C**) Sequencing PCR products show that the WT and *Nlrp3^-/-^* mice have the same downstream sequence (right part of the vertical dotted line, underlined by green band) but different upstream sequence (left part of the vertical dotted line, underlined by black and blue band, respectively). Indeed, the upstream sequence of *Nlrp3^-/-^* mice (underlined by blue band) is the upstream sequence of targeting sites of sgRNA_1_ (see Figure S2A). (**D**) Western blot analysis confirms the loss of full length NLRP3 protein in the BMDM from *Nlrp3^-/-^* mice while strong expression of NLRP3 was detected in WT BMDM. β-actin was used as a housekeeping protein. (**E**) Immunofluorescence analysis shows the lack of NLRP3 fluorescence (green) in *Nlrp3^-/-^* BMDM while strong fluorescence of NLRP3 was observed in WT BMDM. Bars indicate scale of 50μm.
